# Structure of a DNA Glycosylase Bound to a Nicked T:G Mismatch-Containing DNA

**DOI:** 10.3390/molecules30092083

**Published:** 2025-05-07

**Authors:** Hala Ouzon-Shubeita, Rebecca Barnes, Lillian F. Schmaltz, Seongmin Lee

**Affiliations:** The Division of Chemical Biology and Medicinal Chemistry, College of Pharmacy, The University of Texas at Austin, Austin, TX 78712, USA

**Keywords:** DNA damage, DNA glycosylase, mismatch

## Abstract

Mismatched T:G base pairs can arise during de novo replication as well as base excision repair (BER). In particular, the action of the gap-filling polymerase β (Polβ) can generate a T:G pair as well as a nick in the DNA backbone. The processing of a nicked T:G mispair is poorly understood. We are interested in understanding whether the T:G-specific DNA glycosylase MBD4 can recognize and process nicked T:G mismatches. We have discovered that MBD4 binds a nicked T:G-containing DNA, but does not cleave thymine opposite guanine. To gain insight into this, we have determined a crystal structure of human MBD4 bound to a nicked T:G-containing DNA. This structure displayed the full insertion of thymine into the catalytic site and the recognition of thymine based on the catalytic site’s amino acid residues. However, thymine excision did not occur, presumably due to the inactivation of the catalytic D560 carboxylate nucleophile via a polar interaction with the 5′-hydrogen phosphate of the nicked DNA. The nicked complex was greatly stabilized by an ordered water molecule that formed four hydrogen bonds with the nicked DNA and MBD4. Interestingly, the arginine finger R468 did not engage in the phosphate pinching that is commonly observed in T:G mismatch recognition complex structures. Instead, the guanidinium moiety of R468 made bifurcated hydrogen bonding interactions with O6 of guanine, thereby stabilizing the estranged guanine. These observations suggest that R468 may sense and disrupt T:G pairs within the DNA duplex and stabilize the flipped-out thymine. The structure described here would be a close mimic of an intermediate in the base extrusion pathway induced by DNA glycosylase.

## 1. Introduction

**Figure 1:**
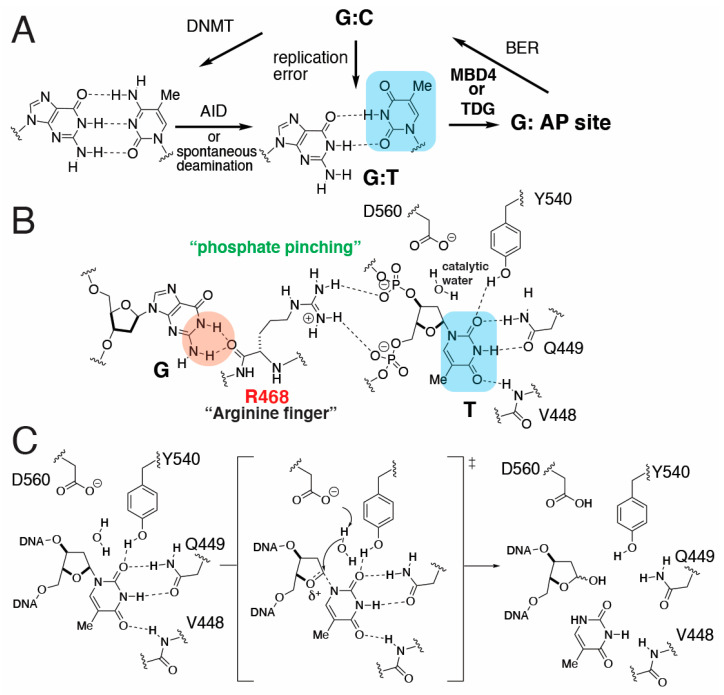
Processing of T:G mismatches by MBD4. (**A**) Formation and base excision repair of T:G mismatches. DNMT: DNA methyltransferase; AID: activation-induced cytidine deaminase; and BER: base excision repair. (**B**) The recognition of flipped-out thymine and orphaned guanine by MBD4. The flipped thymine nucleotide is stabilized via phosphate pinching of the guanidinium moiety of R468. The orphaned guanine is the backbone carbonyl group of R468. The recognition of the Watson–Crick edge of the substrate thymine via catalytic site amino acid residues is shown. (**C**) Glycosidic bond cleavage catalyzed by hMBD4. The nucleophilic water molecule is deprotonated by D560 to generate a hydroxide anion, which in turn cleaves the C-N glycosidic bond of the flipped thymine nucleotide to produce abasic sites.

The maintenance of genome integrity is persistently challenged by the formation of base pair mismatches caused by the deamination of 5-methylcytosine and spontaneous replication errors (Figure 1A). Among the most frequently occurring mismatches is the base pairing between thymine and guanine [1,2], which gives rise to transition mutations if not corrected prior to replication. While T:G adopts a wobble geometry in the absence of protein contacts, T:G base pairs with Watson–Crick-like geometry can be produced in the replicating base pair site of a DNA polymerase that promotes the formation of the rare enol tautomeric or enolate form of T:G [3,4,5,6,7]. For example, the X-family DNA polymerase β (Polβ) and polymerase λ (Polλ) can readily incorporate a thymine nucleotide opposite guanine in the context of a single nucleotide gap [3,8,9,10,11], generating a nicked T:G mismatch-containing DNA.

Methyl-CpG-binding domain protein 4 (MBD4) is a T:G mismatch-specific DNA glycosylase that plays an important role in the maintenance of the methylation states within CpG-repeating regions and the repair of T:G mispairs arising via spontaneous replication error and deamination [12]. MBD4 is a monofunctional DNA glycosylase that belongs to the helix–hairpin–helix (HhH) superfamily of DNA glycosylases [13] with 8-oxoguanine DNA glycosylase 1 (OGG1) and alkyladenine DNA glycosylase (AlkA) [13]. The enzyme is composed of the *N*-terminal methyl-CpG-binding domain (MBD) and the *C*-terminal catalytic glycosylase domain [14]. The known mismatch substrates of MBD4 include uracil, 5-halogenated uracil, hydroxymethyluracil (hmU), and thymine opposite guanine [12,15,16,17,18]. Mammalian MBD4 has been shown to interact with mutL homolog 1 (MLH1), suggesting the role of MBD4 in mismatch repair [15]. The increased mutation frequency at CpG sites of MBD4-/- mice suggests the tumor suppressing properties of the enzyme [14,19,20].

Previous biochemical and structural studies have revealed the mechanisms by which MBD4 cleaves the substrate thymine opposite guanine [14,16,21,22,23,24]. Like other DNA glycosylases, MBD4 induces substrate base flipping [25,26,27] and recognizes the Watson–Crick edge of the flipped thymine (Figure 1B) [28]. The enzyme uses the catalytic aspartate (e.g., D560 of hMBD4 and D534 in mMBD4) as a general base to prime a catalytic water molecule, which excises the C-N glycosidic bond of the thymine via an *S*_N_2 mechanism to generate abasic sites (Figure 1C) [22,23]. During the catalytic process, the estranged guanine engages in hydrogen bonding interactions with the so-called arginine finger (e.g., R468 of hMBD4) [28], which also stabilizes the flipped-out state by creating a “phosphate pinching” mechanism, utilizing its cationic guanidinium moiety. Arginine finger-mediated phosphate pinching is also observed in another T:G mismatch-specific enzyme, thymine DNA glycosylase (TDG) [29].

In our base excision repair studies, we became interested in the fate of a nicked T:G mismatch-containing DNA, a misinsertion product of the gap-filling DNA polymerase Polβ. Can this nicked mispair be recognized and removed by the T:G-mismatch-specific hMBD4? We have found that the nicked T:G-containing DNA is not processed by hMBD4. Herein, we report the high-resolution crystal structure of a catalytically active hMBD4 in complex with a nicked T:G-containing DNA. This structure explains the binding and inactivity of the nicked T:G toward hMBD4 and provides new insights into the role of the arginine finger in the detection of T:G base pairs.

## 2. Results and Discussion

### 2.1. Structure of MBD4 in Complex with a Nicked T:G-Containing DNA

**Figure 2:**
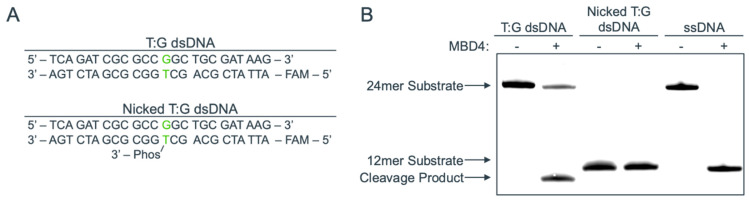
Glycosylase activity assay displaying MBD4’s inability to remove thymine from nicked-T:G–containing DNA. (**A**) DNA sequences used for this assay with the T:G mispair are shown in green for each dsDNA. The ssDNA with the mispaired thymine molecule is FAM-labeled. The nicked T:G dsDNA has a 3′-phosphate attached to the thymine molecule to mimic a nick that could be incorporated by DNA polymerase β. (**B**) A representative gel of duplicate glycosylase activity assays shows that MBD4 can cleave the T:G mispair but cannot cleave the nicked T:G mispair. The 24 mer and 12 mer ssDNA are shown as references for uncleaved DNA.

**Figure 3:**
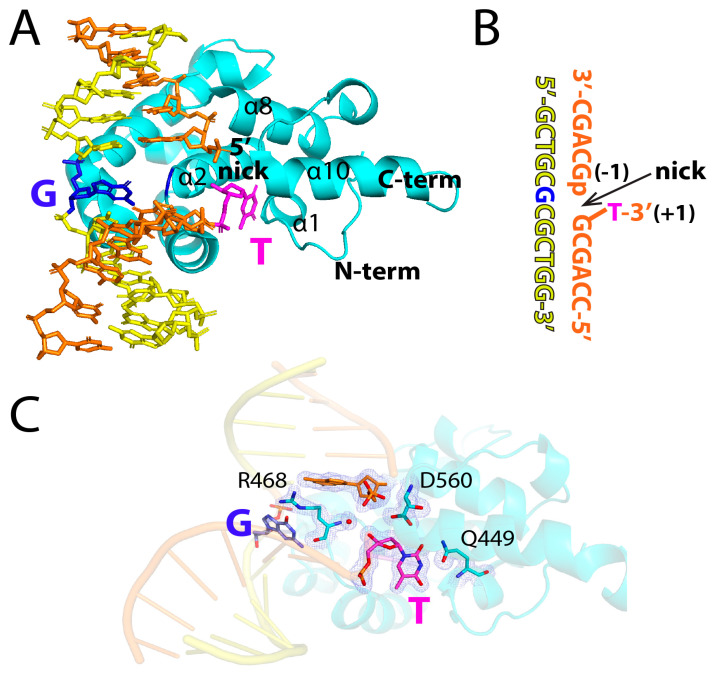
Structure of MBD4 bound to a nicked T:G mismatched DNA. (**A**) The overall structure of a catalytically competent MBD4 in complex with a nicked DNA. Substrate thymine and opposing guanine are colored in magenta and blue, respectively. The nick-containing strand is shown in orange and the complementary strand is shown in yellow. Helices interacting with substrate thymine are indicated. (**B**) The sequence of DNA used for crystallographic studies. (**C**) Overall structure with a 2*F*_o_-*F*_c_ electron density map contoured at 1σ around the flipped thymine nucleotide and the key amino acid residues R449, R468, and D560.

Over the course of our studies on the substrate recognition and glycosylase mechanisms of MBD4, we have found that the catalytic domain of MBD4 does not process a nicked T:G base pair that can arise via misincorporation by Polβ (Figure 2). We expected that a three-dimensional structure of MBD4 in complex with a nicked T:G mismatched DNA would provide insight into the mechanism by which the nicked T:G DNA avoids processing via MBD4. We thus determined the co-crystal structure of hMBD4’s catalytic domain bound to a nicked T:G-containing DNA (Figure 3A), where the nick was introduced at the 3′ side of the thymine opposite guanine (Figure 3B). The nucleotide at the −1 position was 5′-phosphorylated and the thymine opposite guanine had a 3′-OH group to represent a nicked mismatched DNA that can be produced by Polβ. The co-crystal structure, which displayed the *P*2_1_2_1_2_1_ space group, was refined to a 1.8 Å resolution, and the refinement statistics are shown in Table 1.

The overall structure of the MBD4-nicked T:G complex was very similar to that of the published structure of D560N MBD4 bound to T:G mismatched DNA (PDB ID: 4OFA, RMSD = 0.294 Å) [23]. The 2*F*_o_-*F*_c_ electron density map showed the presence of key amino acid residues (e.g., R468, D560, and Q449), G:T mismatches, and the nick (Figure 3C). In the published structure of catalytically inactive D560N MBD4 complexed with a T:G-mismatched DNA, the substrate thymine is flipped out and enters the catalytic pocket of the enzyme, engaging in numerous T:G-specific hydrogen bonds. In our nicked structure, the thymine nucleotide opposite guanine exits the duplex through the major groove and is presented in the catalytically active MBD4, which is created by α1, α2, α8, and α10 helices (Figure 3A). The glycosidic bond of the flipped-out thymine, however, is not cleaved by the wild-type enzyme, indicating that the insertion of thymine into MBD4’s active site does not necessarily lead to the glycosidic bond cleavage.

### 2.2. Binding Mode of a Nicked T:G-Containing DNA to MBD4 and the Origin of Its Inactivity

**Figure 4:**
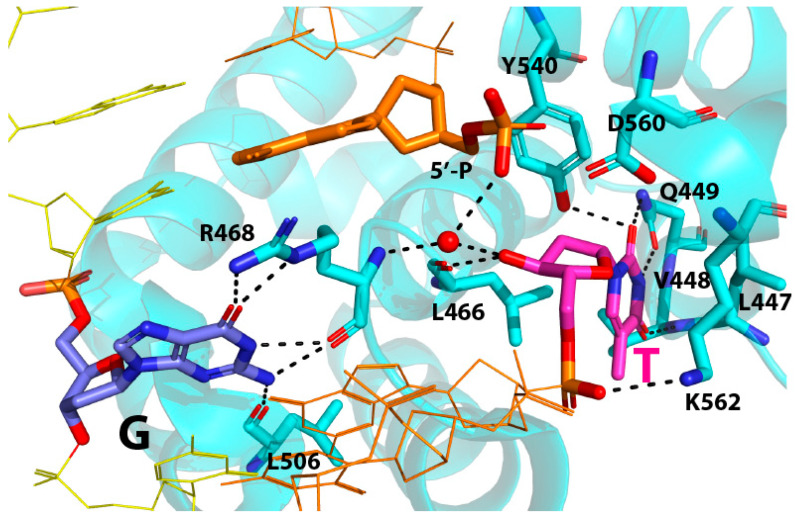
Recognition of the flipped thymine nucleotide by MBD4. Hydrogen bonding interactions near the flipped substrate thymine are shown as dashed lines. Note that Watson–Crick edge of thymine is contacted by V448, Q449, and Y540. Furthermore, 5′-Phosphate of the nicked DNA is hydrogen bonded to carboxylate oxygen of D560 and an ordered water molecule. The ordered water molecule (shown as a red sphere) in turn forms two additional polar interactions with 3′-OH of thymine and R468.

A close-up view of the catalytic site conformation of the nicked T:G-MBD4 complex structure reveals the binding mode of the nicked DNA toward the enzyme (Figure 4). As observed in published MBD4-T:G complex structures [17,21,22,23,24], the target thymine enters the catalytic pocket of MBD4, forming multiple hydrophilic and hydrophobic interactions with catalytic site amino acid residues. All the heteroatoms in the Watson–Crick edge of the thymine engage in hydrogen bonding interactions with V448, Q449, and Y540. Specifically, the O2 and N3 of thymine are hydrogen bonded (2.9 Å and 2.9 Å) to the side chain amide moiety of Q449. The O2 of thymine is also recognized (2.8 Å) by the hydroxyl group of Y540. The O4 of thymine forms a hydrogen bonding interaction (2.8 Å) with the backbone amide moiety of V448. The extruded state of thymine nucleotide is further stabilized by van der Waals interactions with the aliphatic side chains of L447, V448, and L466. The polar contact between the 5′-phosphodiester oxygen of thymine and ε-NH2 of K562 (3.1 Å) also contributes to the stabilization of the flipped thymine nucleotide. Overall, the thymine recognition of the nicked T:G DNA and non-nicked T:G DNA by MBD4 is essentially identical.

**Figure 5:**
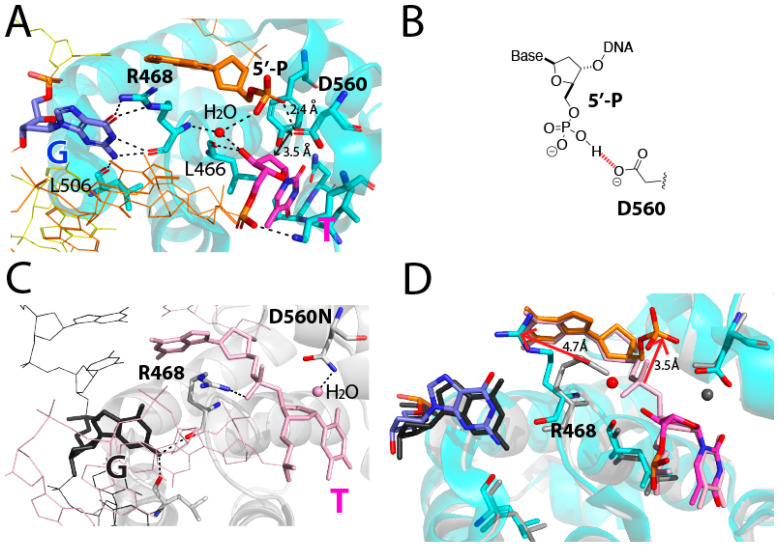
Inhibition of MBD4 via a nicked DNA and the recognition of the orphaned guanine by MBD4. (**A**) The recognition of the estranged guanine by R468 and L506. Hydrogen bonds nearby opposing guanine are shown as dashed lines. Distances between D560 and C1′ of thymine and between D560 and 5′-phosphate oxygen are shown. The ordered water molecule is shown as a red sphere. (**B**) Chemical structures of hydrogen phosphate and D560, where the polar interaction between the two is shown red dashed lines. (**C**) Published structure of MBD4-T:G recognition complex (PDB ID 4OFA). The catalytic water molecule near D560N is shown as a pink sphere. (**D**) Overlay between MBD4-T:G recognition complex (4OFA) and MBD4-nicked T:G complex structures. Major differences are indicated as arrows.

The crystal structure of the nicked T:G mismatch bound to a catalytically competent MBD4 also reveals how this nicked T:G-containing DNA evades the glycosidic bond cleavage by the enzyme. It is believed that MBD4 uses a D560-mediated general base mechanism to perform the glycosylase reaction, where a nucleophilic water molecule is primed by D560 and attacks the C1′ of substrate thymine nucleotide [17,22,23]. In the nicked T:G structure, while the catalytic residue D560 carboxylate is proximal to the C1′ of the extrahelical thymine (3.5 Å, Figure 5A), the nucleophilic water molecule is not present near D560. The lack of the putative water nucleophile in the catalytic site would result in the abrogation of the glycosylase activity of the enzyme. Interestingly, the distance between D560 carboxylate oxygen and the 5′-phosphate oxygen of the nucleotide at the −1 position of the nicked strand was 2.4 Å, indicating a strong hydrogen bonding interaction between them (Figure 5B). Such polar interaction can occur when one of the 5′-phosphate oxygen atoms is protonated to produce hydrogen phosphate. Another 5′-phosphate oxygen engages in polar interaction (3.2 Å) with an ordered water molecule, which in turn forms hydrogen bonds with the 3′-OH of the flipped thymine (2.7 Å) and the backbone amide N-H of R468 (3.0 Å). The 3′-OH of the thymine is hydrogen bonded (2.7 Å) to the backbone carbonyl oxygen of L466. The water-mediated contacts with both the nicked DNA and MBD4 R468 appear to promote the formation of the catalytically incompetent conformation of the nicked T:G-MBD4 complex.

A structural comparison of the nicked complex structure with our published MBD4 T:G mismatch-recognition complex (PDB ID: 4OFA) structure provides insights into the inhibition mechanism of the nicked T:G mismatch (Figure 5C,D). Notably, the 5′ phosphate of the nucleotide at the 3′ side of thymine (or −1 position) moves away from the thymine by ~3.5 Å relative to the position observed in a non-nicked structure (Figure 5C,D), which results in the formation of a polar interaction with D560. This movement would prevent a steric clash between the 5′ phosphate (−1 position) and the 3′-OH group of thymine nucleotide. This conformational reorganization is facilitated by the ordered water molecule that interacts with the two nucleotides at the nick and two active site amino acid residues (R468 and L466). The water molecule in the nicked structure is similarly positioned where the 3′ phosphodiester non-bridging oxygen of the flipped thymine is found in non-nicked structures. While the orientation of the 5′ phosphate at the −1 position is different from that of the corresponding phosphodiester in the D560N MBD4 structure, the orientation of the thymidine nucleotide in the two structures is virtually identical, indicating the occurrence of the full insertion of the substrate thymine into the catalytic pocket. The target thymine recognition by L447, V448, Q449, and Y540 is observed in both structures.

### 2.3. The Role of R468 in T:G Mismatch Recognition

Our crystal structure of hMBD4 in complex with a nicked T:G-containing DNA shows an unusual conformation, where the guanidinium moiety of R468 engages in two hydrogen bonds with the O6 of the estranged guanine, which suggests a new role of R468 in T:G mismatch recognition. In most published MBD4-T:G complex structures, the guanidinium moiety of R468 engages in phosphate pinching by forming hydrogen bonds with a 3′ phosphodiester oxygen of the substrate thymine, thereby stabilizing the extrahelical-positioned thymine (Figure 5C). By contrast, the guanidinium moiety of R468 in the nicked structure makes bifurcated hydrogen bonds with the O6 of the estranged guanine (2.8 Å and 2.8 Å). As the backbone carbonyl group of R468 also makes bifurcated hydrogen bonds with the N1-H and N2-H of the guanine, the combined four hydrogen bonds between the orphaned guanine and R468 in the nicked structure would efficiently replace the two hydrogen bonds typically observed in a T:G base pair with a wobble geometry. The replacement of two inter-base hydrogen bonds of T:G with four hydrogen bonds between G and R468 would readily disrupt the relatively weak T:G base pairing and stimulate thymine nucleotide flipping.

**Figure 6:**
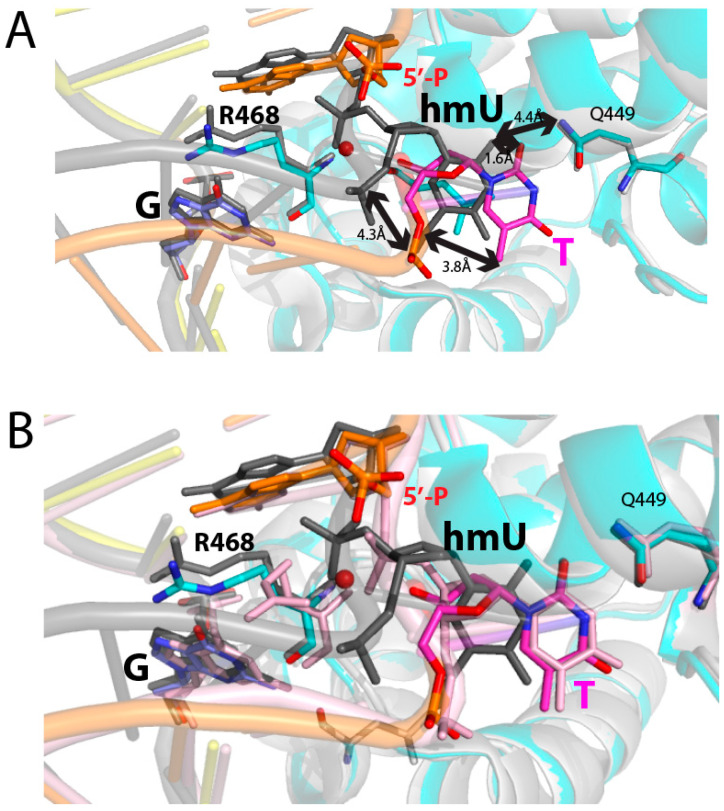
Comparison of the MBD4-nicked T:G, MBD4-hmU:G, and MBD4-T:G structures. (**A**) Superposition of hMBD4-nicked DNA structure (multiple colors) and the D560A hMBD4-hmU:G structure (PDB ID 4EA4, black). Key deviations between the two complexes are indicated as double-headed arrows. The substrate thymine in the nicked complex undergoes full insertion, while hmU in the MBD4-hmU:G structure partially enters the catalytic site. (**B**) Superposition of structures of hMBD4-nicked DNA (multiple colors), the D560A hMBD4-hmU:G (PDB ID 4EA4, black), and D560N hMBD4-T:G (PDB ID 4OFA, light pink) complexes. Note conformational differences in R468 and substrate pyrimidines among these structures.

Interestingly, the R468-mediated recognition of G-O6 has also been observed in a published MBD4 complex structure with a hydroxymethyluracil (hmU):G base pair (PDB ID 4EA4) (Figure 6A), although its structural features have not been described in the paper [16]. In the MBD4-hmU:G structure, the guanidinium moiety of R468 is not making polar contact with the 3′-phosphodiester oxygen of the flipped pyrimidine nucleotide. Instead, it is coplanar to the estranged guanine and makes two hydrogen bonds with G-O6. These interactions would enable R468 to recognize the Watson–Crick edge and major groove of guanine, thereby disrupting the T:G wobble base pair and stabilizing the orphaned guanine. The substrate base hmU flips out but stalls at the entrance of the catalytic pocket in a position that is >4.4 Å away from the catalytic residue D560, forming a non-productive state, as illustrated by the lack of any hydrogen bonding interactions between hmU and active site residues (V448, Q449, and Y540) (Figure 6A). The published MBD4-hmU:G complex structure likely represents an intermediate captured during the base extrusion pathway. In contrast, the nicked T:G-MBD4 structure shows the full insertion of the substrate thymine into the catalytic pocket, while R468 adopts a conformation similar to that observed in the MBD4-hmU:G complex structure.

A comparison of the structures of the MBD4-nicked T:G (PDB ID 5CHZ), D560A MBD4-hmU:G (PDB ID 4EA4), and D560N MBD4-T:G (PDB ID 4OFA) complexes reveals conformational differences in R468, flipped pyrimidines, and substrate-bearing strands (Figure 6B). The D560A MBD4-hmU:G structure depicts a catalytically incompetent state, where R468 interacts with the estranged guanine and the substrate pyrimidine partially enters the catalytic site. The formation of four hydrogen bonds between R468 and the G opposite T would disrupt T:G base pairing, triggering the flipping of the substrate thymine. These structures thus suggest that the final stage of the base extrusion pathway involves a conformational shift in R468 from engaging the orphaned G to stabilizing the substrate T via phosphate pinching, which, in turn, facilitates the full insertion of the flipped thymine into the catalytic site.

Our nicked T:G-MBD4 complex structure provides insights into the potential mechanism by which MBD4 senses T:G mismatches within duplex DNA. We have previously reported that the catalytic activity of MBD4 is greatly influenced by the mutation of R468 [24]. While an R468K MBD4 mutant retains glycosylase activity, the mutation of Arg into Gln, Tyr, Phe, Glu, or Leu results in a complete or near-complete loss of activity [24], suggesting that the guanidinium moiety of R468 and ε-NH2 moiety of R468K may play important roles in MBD4-mediated base excision. Our studies have shown that R468 and R468K residues in two crystal structures (PDB ID 5CHZ and 4OFE) show conformational similarity: R468K engages in water-mediated phosphate pinching, while R468 is involved in direct phosphate pinching. As DNA glycosylases employ a wide range of intercalating residues for phosphate pinching (e.g., Q28 in *Bst*. MutY, L125 of hTDG, Y162 of hAAG, N149 of hOGG1), it is not likely that the lack of activity of several MBD4 R468 mutants is caused by their incapability to stabilize the flipped-base states via phosphate pinching. The analysis of the relationship between the structures of intercalating residues and MBD4 glycosylase activity suggests that a functional group capable of forming hydrogen bond(s) with O6 of guanine and possibly O4 of thymine may be required for the efficient detection of T:G mismatches within the DNA helix. The side chains of Gln, Tyr, Glu, and Leu are less suitable for forming such hydrogen bonds, due to the lack of two hydrogen bond donors and/or the short length of the side chains. By contrast, the side chains of Lys and Arg are relatively long and can provide two hydrogen bond donors to the G-O6 and T-O4 hydrogen bond acceptors. We speculate that MBD4 uses R468 to sense T:G mismatches within duplex DNA prior to triggering thymine flipping.

### 2.4. The Role of R468 in the Base Extrusion Pathway

The computational and structural studies of various DNA glycosylases bound to their substrate DNA have suggested that base extrusion occurs through multiple conformational intermediates rather than occurring through a single concerted step [30,31,32,33,34]. For example, structural studies of hOGG1 in complexes with C:G or C:oxoG base pairs result in the capture of base extrusion intermediates. These structures have revealed how the enzyme recognizes the lesion inside the DNA helix and triggers the base extrusion pathway [31,32]. A disulfide cross-linking strategy and a photocaging approach have led to the capture of several structural intermediates of the base extrusion pathway [33,34]. The structural similarity between our nicked structure and the MBD4-hmU:G structure (Figure 6) suggests that these unusual conformations may mimic a conformational intermediate that arises during the base extrusion pathway. These structures also suggest that R468 plays a critical role in the recognition and disruption of T:G mismatch and the stabilization of base extrusion intermediates. They imply that, during the MBD4-induced base extrusion pathway, the stabilization of the estranged guanine via R468 occurs first. Then, the phosphate pinching-mediated stabilization of the flipped thymine takes place at the final stage of the base extrusion pathway, causing full insertion of the substrate thymine into the catalytic pocket of MBD4.

The recognition of estranged nucleobases is crucial for DNA glycosylases that must recognize specific base pairs [35,36,37]. For alkyladenine DNA glycosylases such as hAAG and *E. coli* AlkA, the estranged nucleobase is devoid of any protein contacts, as these enzymes process alkylated purines irrespective of opposite bases [27,38]. By contrast, base pair-specific DNA glycosylases such as OGG1, MutY, and TDG cleave oxoG opposite cytosine, adenine opposite oxoG, and thymine opposite guanine, respectively. In these DNA glycosylases, the removal of nucleobases in different base pair contexts, such as the cleavage of oxoG opposite adenine by hOGG1, leads to a mutagenic repair. Crystal structures reveal how these enzymes discriminate substrate base pairs against non-substrate base pairs. In the case of hOGG1, the Watson–Crick edge of the orphaned cytosine is recognized by the guanidinium moieties of R204 and R154 and the side chain amide moiety of N149 [39]. In the case of *Bst* MutY, both the Hoogsteen and Watson–Crick edges of the estranged oxoG are recognized by Q48, T49, L86, and S308 of *Bst* MutY [40]. In the case of the T:G mismatch-specific DNA glycosylase hTDG, the opposing guanine is contacted by the backbone carbonyl oxygen of A274 and P280 [29]. Like hMBD4, hTDG has an arginine finger R275, but this residue does not participate in guanine recognition. R275 engages in the phosphate pinching of the flipped thymine, instead. Lastly, in the case of another T:G mismatch-specific DNA glycosylase hMBD4, the alienated guanine is contacted by the arginine finger R468, which also stabilizes the flipped thymine nucleotide via phosphate pinching.

## 3. Materials and Methods

Oligonucleotides used for biochemical and structural studies: Modified oligonucleotides were purchased from Midland Certified Reagents (Midland, TX, USA). The nicked DNA sequences used with wild-type MBD4 were 12-mer oligonucleotide 5′-GCTGCGCGCTGG-3′, 7-mer upstream primer 5′-CCAGCGT-3′, and a 5-mer downstream primer 5′-phosphate-GCAGC-3′. The oligonucleotides used for the glycosylase activity assay were purchased from Integrated DNA Technologies (Coralville, IA, USA). The 24 mer sequences used for the T:G mispair dsDNA (no nick) were 5′-TCAGATCGCGCCGGCTG CGATAAG-3′ and 5′-FAM-CTTATCGCAGCTGGCGCGA TCTGA-3′. For the nicked T:G dsDNA, the same 24 mer with guanine was used, along with two 12 mers, one with 3′-phosphate-thymine and FAM labeled, and the other unmodified. These sequences were 5′-FAM-CTTATCGCAGCT-Phos-3′ and 5′-GGCGCGATCTGA-3′, respectively.

Protein expression and purification of MBD4 glycosylase domain: The gene of the human MBD4 glycosylase domain with residues 425–580 was amplified via PCR and inserted into the pET28a vector. The MBD4 glycosylase domain was expressed using the pET28a vector. The proteins were expressed in *E. coli* BL21 (DE3) cells. The cultures were grown in Luria–Bertani (LB) broth medium at 37 °C until the OD_600_ of 0.5, where the cells were induced with 0.25 mM of isopropyl β-D-α-thiogalactopyranoside (IPTG) for four hours at 37 °C. Pelleted cells were resuspended in buffer (50 mM sodium phosphate, pH 7.8, 500 mM NaCl, 10% glycerol, 1 mg/mL lysozyme, 0.25% NP-40, 0.25% Triton X-100, and 0.25 mM phenylmethylsulfonyl fluoride) and sonicated for 30 s. The lysate was centrifuged at 15,000× *g* at 4 °C for 20 min. The MBD4 glycosylase domain protein supernatant was purified using Ni–NTA column (GE Healthcare, Chicago, IL, USA). The imidazole-eluted fractions from the Ni-NTA column were combined and buffer-exchanged to low imidazole buffer, treated with thrombin (10 units), and incubated for 16 h at 4 °C. This removed the 6-His tag, thereby leaving 19 extraneous N-terminal amino acids (GSHMASMTGGQQMGRGSEF). The cleaved proteins were further purified on Superdex 75 column (GE Healthcare) equilibrated with a buffer (50 mM Tris, pH 7.5, 150 mM NaCl, and 10% glycerol).

DNA glycosylase activity assay: The glycosylase activities of MBD4 were performed using various FAM-labeled oligonucleotides. The glycosylase activities of MBD4 R468 mutants were performed using 27-mer, 5′-FAM-labeled oligonucleotide duplex containing a G:T mismatch in a CpG region (5′-TCAGATCGCGCC **G**GCTGCGATAAGCT-3′ and 3′-AGCTTATCGCAGC**T**GGCGCGAATCTGA-FAM-5′; T:G mismatch is indicated in bold and underlined), and the corresponding nicked DNA (5′-TCAGATCGCGCC**G**G CTGCGATAAGCT-3′, 3′-AGCTTATCGCAGC-5′-phosphate, and 3′-**T**GGCGCGAATCT GA-5′-FAM). The standard reaction mixture (20 μL) contained 50 nM of labeled double-stranded oligonucleotide and 2.5 uM of purified enzyme in 20 mM HEPES pH 7.5, 50 mM KCl, 1 mM DTT, 1 mM EDTA, and 0.1 mg/mL bovine serum albumin. The standard reaction mixture (20 µL) contained 50 nM of labeled double-stranded oligonucleotide and 5 µM of purified enzyme in 20 mM HEPES pH 7.5, 50 mM KCl, 1 mM DTT, 1 mM EDTA, and 0.1 mg/mL of bovine serum albumin. The mixture was incubated at 37 °C for 30 min, and the reaction was stopped by adding 4 µL of 1 N NaOH followed by boiling at 70 °C for 10 min to cleave DNA at the abasic site. After adding loading dye (5 mL, 98% formamide, 1 mM EDTA, 1 mg/mL of Bromophenol Blue, and 1 mg/mL Xylene Cyanole), the reaction sample was loaded onto a 10 cm × 10 cm 20% denaturing urea gel. The FAM-labeled DNA intensities were measured using Typhoon FLA9500 (GE Healthcare).

Protein-DNA co-crystallization: A 12-mer crystallization DNA was annealed at a 1:1 ratio and mixed with the protein (110 μM) in a 1:5 molar ratio of protein to DNA in a buffer containing 5 mM Tris pH 8.0 and 25 mM NaCl. The DNA–protein mixture was incubated on ice for two to three hours. The wild-type MBD4-nicked DNA complexes were crystallized in 21% ethylene glycol. The hanging drop method was used for crystallography by mixing 1 μL of protein–DNA complex solution with 1 μL of reservoir solution. Crystals of MBD4 were grown at 22 °C over two weeks. The crystals were flash-frozen in liquid N_2_ and used for data collection.

**Data collection and structure determination**: Diffraction data sets for crystals of the MBD4 complexes were collected at a wavelength of 0.97948 Å on beamline 5.0.3 at the Advanced Light Source (Berkeley, CA, USA). Diffraction data for the MBD4 complexes were indexed and scaled using HKL2000. The MBD4-DNA complex structures were solved via molecular replacement using an MBD4-DNA complex (Protein Data Bank accession code 4E9G) as the search model. Manual model building was carried out with COOT and refined using CCP4 and REFMAC [41,42,43]. The quality and stereochemistry of the models were examined with PROCHECK. All the crystallographic figures were prepared using PyMOL. The statistics of data processing, data quality, and the refined models are summarized in Table 1.

## Figures and Tables

**Table 1:** Data collection and refinement statistics.

PDB Code	wt MBD4-Nicked DNA (5CHZ)
**Data Collection**	
Space Group	*P*2_1_2_1_2_1_
Cell constants	
a (Å)	41.886
b	54.875
c	104.522
Resolution (Å) ^a^	39–1.83 (1.89–1.83)
*R*_merge_ ^b^	0.071 (0.544)
<I/σ>	30.7 (3.15)
Completeness (%)	97.7 (96.8)
Redundancy	6.9 (6.8)
**Refinement**	
*R*_work_^c^/*R*_free_^d^ (%)	18.6/21.8
Unique reflections	20,686
Mean B factor (Å^2^)	
Protein	26.65
Ligand	17.97
Solvent	36.02
Ramachandran plot	
Most favored (%)	99.3
Additional allowed (%)	0.7
RMSD	
Bond lengths (Å)	0.018
Bond angles (°)	1.81

^a^ Values in parentheses are for the highest resolution shell. ^b^ *R*_merge_ = Σ|I − <I>|/ΣI, where I is the integrated intensity of a given reflection. ^c^ *R*_work_ = Σ|F(obs)-F(calc)|/ΣF(obs). ^d^ *R*_free_ = Σ|F(obs)-F(calc)|/ΣF(obs), calculated using 5% of the data.

## Data Availability

The atomic coordinate of the MBD4-DNA complex has been deposited in the Protein Data Bank with the accession code of 5CHZ.
